# Sex/Gender-Specific Imbalance in CVD: Could Physical Activity Help to Improve Clinical Outcome Targeting CVD Molecular Mechanisms in Women?

**DOI:** 10.3390/ijms21041477

**Published:** 2020-02-21

**Authors:** Mauro Vaccarezza, Veronica Papa, Daniela Milani, Arianna Gonelli, Paola Secchiero, Giorgio Zauli, Donato Gemmati, Veronica Tisato

**Affiliations:** 1 School of Pharmacy and Biomedical Sciences, Faculty of Health Sciences, Curtin University, Bentley, Perth, WA 6102, Australia; 2 Department of Motor Sciences and Wellness, University of Naples “Parthenope”, 80132 Napoli, Italy; veronica.papa@uniparthenope.it; 3 Department of Morphology, Surgery & Experimental Medicine and LTTA Centre, University of Ferrara, 44121 Ferrara, Italy; daniela.milani@unife.it (D.M.); arianna.gonelli@unife.it (A.G.); paola.secchiero@unife.it (P.S.); giorgio.zauli@unife.it (G.Z.); veronica.tisato@unife.it (V.T.); 4 Department of Biomedical & Specialty Surgical Sciences, and Centre Haemostasis & Thrombosis, University of Ferrara, 44121 Ferrara, Italy; cet@unife.it; 5 Centre of Gender Medicine, University of Ferrara, 44121 Ferrara, Italy

**Keywords:** CVD, sex/gender differences, clinical outcome, physical activity, molecular mediators

## Abstract

In the last two decades, new insights have been gained regarding sex/gender-related differences in cardiovascular disease (CVD). CVD represents the leading cause of death worldwide in both men and women, accounting for at least one-third of all deaths in women and half of deaths in women over 50 years in developing countries. Important sex-related differences in prevalence, presentation, management, and outcomes of different CVDs have been recently discovered, demonstrating sex/gender-specific pathophysiologic features in the presentation and prognosis of CVD in men and women. A large amount of evidence has highlighted the role of sex hormones in protecting women from CVDs, providing an advantage over men that is lost when women reach the menopause stage. This hormonal-dependent shift of sex-related CVD risk consequently affects the overall CVD epidemiology, particularly in light of the increasing trend of population aging. The benefits of physical activity have been recognized for a long time as a powerful preventive approach for both CVD prevention and aging-related morbidity control. Exercise training is indeed a potent physiological stimulus, which reduces primary and secondary cardiovascular events. However, the underlying mechanisms of these positive effects, including from a sex/gender perspective, still need to be fully elucidated. The aim of this work is to provide a review of the evidence linking sex/gender-related differences in CVD, including sex/gender-specific molecular mediators, to explore whether sex- and gender-tailored physical activity may be used as an effective tool to prevent CVD and improve clinical outcomes in women.

## 1. Introduction

Sex/gender-related differences in cardiovascular disease (CVD) risk, clinical phenotype, and outcome have been discovered in the past two decades [1]. These new concepts have an important translational value as CVD is the leading cause of death in older people and there is a global increase in the elderly population. Notably, almost 60% of elderly patients with atherosclerotic CVD have either no conventional risk factors or just one [2].

Of particular relevance is the issue of heart failure, which is expected to double each decade of life and to grow significantly in the whole population due to the dramatic increasing trend of population aging, particularly in the developed countries [3]. Although several novel molecular markers and pharmacogenetic studies have been used to intensively investigate complex polygenic chronic or degenerative diseases [4,5,6,7,8,9,10,11,12,13] leading to the discovery of novel prognostic biomarkers or inherited predispositions [14,15,16,17,18,19], specific dedicated therapies to treat heart failure due to heart wall remodeling do not currently exist, and women experience the worst prognosis [1,20,21]. Epidemiological data highlight that CVD now represents the leading cause of mortality and hospital admission for women, accounting for one in three deaths worldwide and half of all deaths of women over 50 in developing countries [3]. By contrast, breast cancer, considered in the past to be the leading cause of death in women, now accounts for just 3% of all deaths in the female adult population [1,22]. Several studies and reports have unequivocally demonstrated the key role of sex hormones in protecting women from CVD during the premenopausal state, providing an advantage over men that is lost when the hormonal profile changes at menopause transition. This hormonal-dependent shift of sex-related CVD risk strongly affects CVD epidemiology and CVD prevention/treatment, particularly in light of the aging of the overall population [20,23]. These clinical data suggest that novel risk factors that are still not well established may contribute to the development of CVD in a sex- and gender-dependent fashion.

In real practice, new therapeutic treatments useful to men have not led to a significant decrease in CVD fatality rates in women [1,24], supporting the key role of sex/gender differences in determining CVD risk, diagnosis, and prognosis and explaining the existing gap in the progress achieved to treat CVD in women compared to men [25]. The emerging view is that disparities in knowledge regarding CVD in women over men can be overcome through a sex/gender-based “holistic” approach to disease pathophysiology [1,20], spanning from clinical signs and manifestations up to risk establishment, diagnosis definition, and dedicated treatment design through appropriate clinical trials in which the role of sex and gender differences can be properly evaluated in terms of therapeutic response [22]. In fact, although milestone studies in the last few decades have allowed the development/optimization of actual standard care in the context of CVD by taking advantage of more accurate clinical tests that have allowed a better definition of the risks and benefits of effective prevention/therapies, the majority of these studies did not appropriately consider women. Thus, much of the actual standard care available to test, prevent, and treat CVD in women can be considered a mere translation of the findings of studies conducted predominantly on middle-aged men [3], an approach that is inappropriate and risky given the emerging role of sex/gender differences in CVD pathophysiology. To apply the findings of studies on male cohorts to females for the management of CVD may be inappropriate for several reasons, including dissimilarities in symptoms of CVD, natural disease history, and response to therapies that are different in men and women [26]. A proper consideration of sex/gender-related differences in medicine and particularly in the evaluation of occurrence, risk evaluation, presentation, therapy, and outcomes of CVD is a recent achievement [3]. It is now widely recognized that there is an incremental shift in how CVD should be treated in women. This has left healthcare professionals unprepared, mainly because of the poor understanding of sex/gender-specific differences in the pathophysiology of CVD, resulting in a dramatic absence/insufficiency of diagnostic and therapeutic guidelines to address personalized differences in women [1,3].

A paradigmatic example of sex differences in CVD is the clinical manifestation and symptom presentation, with women more likely to show ischemia with no obstructive coronary arteries (INOCA), although they report more chest pain compared to men [27,28,29,30]. However, sex differences may also have a different impact on traditional CVD risk factors. Diabetes is associated with higher ischemic heart disease (IHD) in women compared to men, even if risk factors are exclusive to women (e.g., pregnancy-related complications) or they mainly disadvantage women (e.g., depression) [30]. In addition, comorbidities, such as psychological factors, vasoconstriction, and microcirculatory dysfunction during mental stress, are phenomena highly prevalent in patients with angina and more pronounced in women [31].

The current view clearly suggests a multifactorial model, with sex hormones playing a crucial role by interacting with traditional and nontraditional risk factors, in turn affecting atherosclerotic plaque deposition and/or vascular/metabolic pathways and resulting in poorer outcomes in women [32]. Furthermore, although the major CV risk factors are the same in both sexes, sex/gender-specific biases have been recognized [33], and these differences are related to different outcomes. Data from the Framingham Heart Study by Kannel and co-workers demonstrate that the clinical manifestation of atherosclerotic CVD is extremely rare in younger women compared to men of similar age. The analysis of CVD incidence suggests that premenopausal women have fewer CV and coronary events compared to men as well as postmenopausal women of the same age, even if there is also substantial sex-related variability in the prevalence/outcome associated with traditional cardiac risk factors, such as family history of premature coronary heart disease (CHD), cholesterol levels, hypertension, smoking, or obesity [34,35].

Studies aimed at precision medicine are providing increasing data highlighting that it is indeed no longer possible to avoid a proper consideration of sex/gender differences when approaching disease pathophysiology [36,37,38]. Environmental, social, and psychological differences can contribute to sex/gender imbalances in CVD. In the same fashion, sex hormones account for emerging sex-related differences in CVD, as elucidated by recent investigations of molecular mechanisms [39,40]. Newly identified risk indicators, including inflammatory markers such as high-sensitivity C-reactive protein, IL-6, fibrinogen, and acute phase protein as well as other pathophysiological processes such as retinal artery narrowing, coronary artery calcification, endothelial dysfunction, and anemia, are now being studied in women [41]. Finally, the gut microbiome, harboring trillions of microbial cells, is gaining increasing interest as it has been demonstrated to play an important role in the development of CAD [42,43]. In this context, Li and colleagues recently reported that plasma levels of trimethylamine N-oxide, a metabolite derived from precursors of diet originating from gut microbiota activity, was a prognostic biomarker of major adverse cardiovascular event (MACE) risk in short- and long-term follow-up [44]. Moreover, the growing interest in the role of microbiome in disease pathophysiology has led to the discovery of microbiome-related sex differences that have been suggested to be involved in pathological contexts, such as inflammation, autoimmunity, cardiometabolic diseases, and depression [45,46]. Sex differences in microbiome belong to different causes, including (but not limited to) different genetic background, diverse energy balance, and dietary needs across the lifespan as well as differences in gastrointestinal transit time [47].

Differences between men and women in terms of effects of physical exercise have been reported, and sex/gender-specific studies are gaining increasing attention and highlighting crucial implications in several health issues, including cardiac adaptation to exercise as well as CVD prevention/rehabilitation [48,49,50,51,52,53,54].

In this scenario, the aim of the present review is to critically discuss key evidence related to sex/gender-related differences in CVD and examine the beneficial role of physical activity to support a potential role of sex/gender-tailored physical exercise to target CVD physiopathological processes and improve clinical outcomes, particularly in women, by highlighting the key molecular and cellular mediators involved.

## 2. Women-Specific CVD Risk Factors and Their Molecular Mechanism

CVD has long been considered a male-specific disease even though CVD also affects women, although later in life than in men. There is also a misperception that CVD among women is not as severe as it is in men [55], despite evidence of a progressive increase in CVD in women as demonstrated by epidemiological data.

In 2016, Garcia and colleagues extensively studied the unique aspects of CV health in women [56] and provided an in-depth analysis of sex and gender differences relating to clinical practice in the prevention, diagnosis, and treatment of CVD. One of the main differences in the prevalence of CVD in women, particularly in postmenopausal women, is certainly the effect of estrogen in the regulation of vascular endothelium and the cardiovascular system. According to Sciomer and co-workers, sex/gender disparities in cardiology also stem from biological differences, including endothelial cell dysfunction processes [23]. The role of nitric oxide (NO) in physiological endothelial cell biology/homeostasis as well as in the pathophysiological processes underlying atherosclerosis and vascular disease has been widely investigated, as reviewed in [57]. In terms of sex-related differences in CVD, it is of great relevance that estrogen also modulates endothelial functions by increasing the expression and activity of endothelial NO synthase (eNOS) by genomic and nongenomic mechanisms [58]. In particular, by interacting with estrogen receptor-α (ΕRα), estrogen modulates endothelial and vascular smooth muscle cell functions via the mitogen-activated protein kinase (MAPK) pathway [39]. In this context, the estrogen decline characterizing menopause (as well as the presence of alterations in ERα) is coupled with impaired NO-mediated effects, including reduced endothelial healing/angiogenesis/vasodilatation and altered inflammatory status [59].

Together with changes in the hormonal profile, the higher risk and occurrence of CVD experienced by postmenopausal woman can also be related to additional (perhaps synergic) causes, including differences in response to emotional/physical stress and/or sex- and gender-specific vascular and metabolic effects [42,60,61]. In the same fashion, the complex cascade mediated by estrogen may lead to changes in coagulation and other systems, accounting for the key role of sex hormone fluctuations in establishing CVD risk, including those occurring during menopause and pregnancy [39,62]. A recent study by Baetta and co-workers [37] clearly reviewed the current status of studies investigating the molecular basis of sex differences in CVD, mainly through the proteomic approach. In this regard, studies on the circulating levels of potential soluble CVD markers have revealed sex-specific differences, such as those in serum levels of the adipocyte fatty acid-binding protein (A-FABP), emerging as a relevant atherosclerosis marker in women (i.e., with different fat distribution and hormonal regulation compared to men), supporting the need for sex/gender-specific biomarkers to be translated in clinical practice [63,64]. Accordingly, it has been recently demonstrated that females with angina are more thrombogenic than males, and this inevitably affects clinical outcome and prognosis [65]. Important sex differences have been identified in fat distribution, with women typically showing peripheral subcutaneous adipose tissue, while men tend to accumulate more central adipose and visceral adipose tissue [26]. These various findings may have a great role in establishing sex- and gender-related differences in CAD risk because low endothelial shear stress promotes the development of atherosclerosis [66] and it has been associated with fat accumulation/distribution, endothelial wall remodeling, inflammation, and plaque instability [67,68].

Particularly with regard to endothelium modifications and their relationship to CVD, in 1994, Celermajer and colleagues [69] published data from 103 men and 135 women with no risk factors for atherosclerosis. The results indicated that artery flow-mediated dilatation (FMD)—an index of endothelial functionality response to increased luminal shear stress, at least in the NO-mediated part [70] that predicts cardiovascular events [71,72,73]—declined in men at an earlier age than in women. The authors also noted a steep decline of artery FMD in women that coincided with the time of menopause [69]. In 1995, Hashimoto and colleagues [74] were the first to evaluate vascular endothelium changes during the menstrual cycle. They highlighted that FMD was equal to that of males at low circulating estradiol levels, while FDM responses were increased in the presence of high estradiol (i.e., follicular phase) [74]. These data have been confirmed by several independent laboratories, including the recent report by Williams and co-workers [75]. A number of different studies have recently investigated the molecular mechanisms associated with the impacts of 17β-estradiol on vascular functions and endothelium remodeling, suggesting that these adaptations, which include the generation of NO and prostacyclin, possibly act through the promotion of endothelial repair and regeneration via the production of anti-inflammatory and antioxidant agents depending on the receptor subtype [76,77].

The role of estrogen receptors is indeed crucial in the atherosclerotic process where, in addition to the classical estrogen receptors (i.e., ERα and estrogen receptor-β (ERβ) shown to mediate opposite effects), other receptors may be involved, suggesting that the balance between the different ERs may be responsible for the different responses and effects [23,78]. Moreover, a very recent study on an adult zebrafish model demonstrated that the heart strongly responded to 17β-estradiol in a sex-specific manner, suggesting that zebrafish could be used as a model to identify novel sex-targeted cardiovascular therapies [79]. Finally, a recent study by Tarhouni and co-workers [80] demonstrated an essential role of 17β-estradiol and ERα in flow-mediated remodeling of resistance arteries, suggesting potential synergic impacts of endothelial shear stress and estrogen signaling in terms of vascular adaptation following exercise and training in vivo. Notably, vascular stiffness is a recognized CVD risk factor with sex-specific differences and mechanisms, showing a superior role in postmenopausal women affected by CVD (e.g., preferential heart failure with preserved ejection fraction or isolated systolic hypertension) [81].

## 3. Impact of Exercise Training on CVD Risk

**Figure 1:**
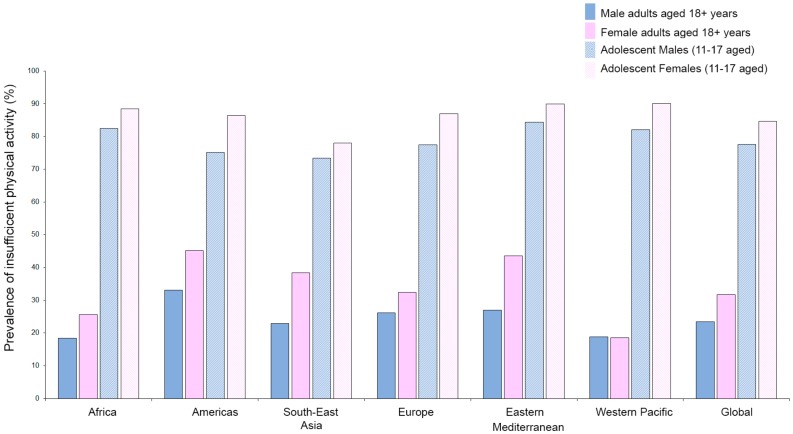
Prevalence of insufficient physical activity as reported by WHO (https://www.who.int/gho/ncd/risk_factors/physical_activity/en/; data from 2016) in adults (aged 18+ years) and adolescents (11–17 years) stratified by sex and region.

Exercise training is recognized as a powerful stimulus that is able to control and decrease primary [82,83] and secondary cardiovascular events [84]. However, as reported by the World Health Organization (WHO), about 23.4% of adult males and 31.7% of adult females (aged over 18 years) are not active enough (2016 data). The crisis is even more severe when considering the fact that 81% of adolescents aged 11–17 years are not active enough and young girls (88.4%) are significantly less active compared to their age-matched young male counterparts (82.4%). Furthermore, stratifying by regions, women are generally less active than men (with the exception of the Western Pacific region), and young adolescent girls are at a higher risk of developing noncommunicable diseases (e.g., heart disease, stroke, cancer, diabetes, and chronic lung disease) and death (Figure 1) [85].

**Figure 2:**
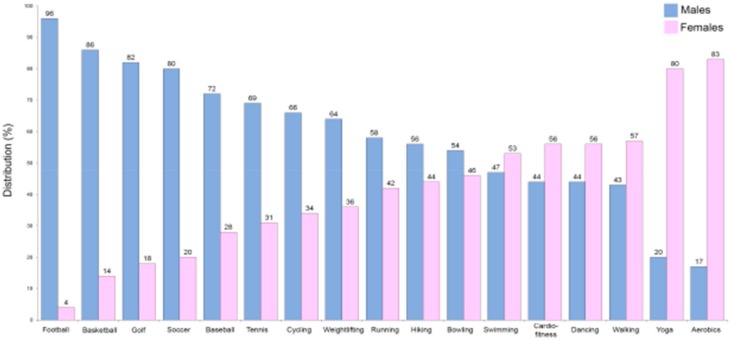
Comparison of common sports engaged in according to sex in the US (data from the American Time Use Survey, https://www.bls.gov/spotlight/2008/sports/; The Bureau of Labor Statistics (BLS)).

An excessively high level of physical inactivity was also reported in the Eurobarometer on Sport and Physical Activity of the EU in March 2018 [86]. Insufficient physical activity increases the risk of CVD, heart disease, cancer, stroke, and diabetes by 20%–30% and shortens the lifespan by 3–5 years [85]. Exercise improves artery and muscle functions [87] and seems to be of great importance in the prevention/modulation of a number of atherogenic processes [88]. Therefore, it is likely that due to improvements in vascular functions, exercise and physical activity could be linked to reduction in CVD [89]. In particular, considering that the worse CVD prognosis in females is partly due to the nonoptimized therapies they receive as described above, an improvement in time/quality of physical exercise could be a way to bridge this gap between the sexes. The fact that males have historically been more likely to be involved in specific sports/physical activities that are different from those practiced by females must also be taken into account (Figure 2).

Tinker and co-workers [83] were the first to investigate the protective effects of vascular remodeling during exercise in healthy subjects. They demonstrated that an increase in FMD following an acute bout of handgrip exercise was dependent on increased shear stress and that functional and remodeling vascular adaptations to training were therefore dependent on shear stress. Moreover, they defined exercise training as providing a potent physiological stimulus to adaptation in endothelial function and vascular remodeling, noting that this particular shear stress in response to exercise training in healthy humans is the principal mechanism responsible for exercise-induced vascular adaptations in function and structure [83].

**Figure 3:**
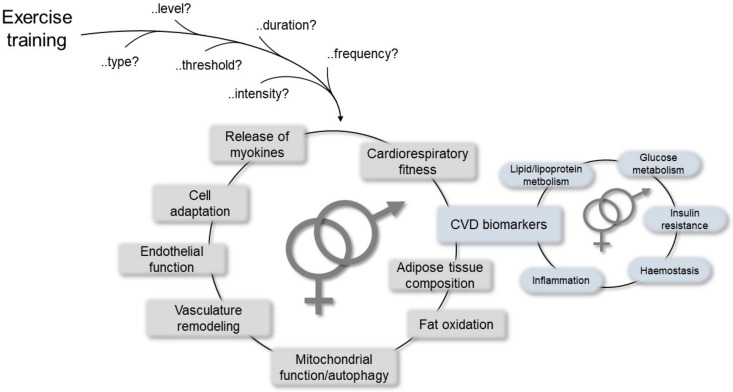
Schematic representation of the most relevant mechanisms mediating cardiovascular (CV) responses/improvements due to physical exercise, which may help to establish differences between the sexes.

Many studies have reported significant inverse association of physical activity with mortality and CVD morbidity [61]. In particular, in a systematic review and meta-analysis, Lin and colleagues clearly indicated that exercise training improved cardiorespiratory fitness and cardiometabolic biomarkers belonging to different biological pathways, such as lipid/lipoprotein metabolism, glucose intolerance, insulin resistance, and systemic hemostasis/inflammation, thereby highlighting sex as one of the variables that may differentiate the effects and benefits of exercise at the CV level together with age and health status [90]. Other advantages have also been reported, including positive effects on mitochondrial function, re-establishment of vasculature, release of myokines from the skeletal muscle that preserve or augment cardiovascular functions [91], and mitochondrial selective autophagy in the view of the crucial role of mitochondria in maintaining cardiac homeostasis (Figure 3) [92].

It is worth noting that different consideration and attention should be paid to different types of physical activities. In particular, leisure/recreational activities and occupational-related activities (e.g., housework, transportation, lifting heavy loads) should be distinguished, with the latter being less proven (up to now) by epidemiological studies in terms of disease prevention/risk establishment [93].

In a recent study published in the *Lancet*, Lear and colleagues [94] recruited 168,916 participants from 17 countries, 141,945 of whom completed the study, to investigate whether different levels and types of physical activity are associated with different outcomes in terms of mortality and CVD in countries at different socioeconomic levels [94]. During follow-up, there were 5334 total deaths, including 1294 from CVD causes and 4040 deaths from non-CVD causes. Results suggested that high physical activity was associated with lower CV risk for all outcomes independent of the type of physical activity (i.e., recreational or nonrecreational). In addition, the association between any type of physical activity and a lower CVD risk (e.g., mortality and major events) was independent of the presence of other risk factors [94].

In addition to physical/CV effects, the positive benefits of physical exercise have also been recognized at the emotional, social, and psychological levels. However, the fact that people of different sex/gender may need different types/requirements (e.g., intensity and duration) of physical exercise is rarely considered. In fact, while there is a general agreement on the benefits obtained by both sexes when moving from sedentary to moderate physical activity [95], reports in the field appear to suggest that more intense physical exercises may lead to different outcomes in men and women [96,97,98]. In particular, the impact of a shift from moderate to strong/energetic physical training appears to mainly favor men, while women seem to gain benefits from low to moderate exercise with no additional positive outcomes when increasing the intensity, particularly among postmenopausal women [99]. With regard to cardiovascular health outcomes, low/moderate physical activities have been reported to be more protective in women in terms of CVD risk, coronary heart disease, and other pathological conditions, such as diabetes [49,97,100,101,102,103,104]. However, few studies have so far compared the response to different types of exercise in women and men in the prevention of CVD risk and during rehabilitation [49,105,106,107,108,109]. It is of interest that recent studies on the effects induced by endurance and endurance–strength exercise in women characterized by obesity revealed that not only were there exercise-mediated improvements in terms of renal function [110], liver function [111], and mineral homeostasis [112] but that the type of exercise could make a difference in terms of biochemical parameter markers of exercise-mediated benefits [111].

In terms of mechanistic insights, these sex-related differences may be obviously attributed to anatomical/biological cardiovascular differences between men and women, such as heart rate during physical exercise, size and number of red cells, and airway and lung development, just to cite few paradigmatic parameters that may significantly affect the response and performance to a specific type of physical training. Similarly, sex-specific differences, also linked to natural intrinsic differences and sex-specific thresholds, have been reported with regard to benefits from both aerobic exercise and resistance training, with women showing a lowering of diastolic pressure following resistance training while men showed benefits in terms of increase in arterial stiffness, suggesting the need for sex-specific exercise as intervention [113].

Sex-related differences have also been investigated at the preclinical level in animal models to evaluate the role of sex hormones and other molecular mediators, such as NO and myosin heavy chain expression, on the regulation mediated by physical activity [114,115]. Interestingly, a recent study by Rosenfeld [116] investigated sex-related differences in physical activity levels in both rodents and humans, highlighting the brain regions and the signaling pathways that are most likely involved. Overall, preclinical and human subject studies support the evidence of sexually dimorphic differences in physical activity. More specifically, animal studies suggest that, under basal conditions, females tend to be more active than males [117], whereas human males are more active than females [118,119]. It is of interest that human males tend to have more intrinsic motivators linked/leading to physical activity, such as the need/desire to improve health quality, prevent disease onset, and improve body shape as well as being competitive. On the contrary, females of different ages appear to be motivated to undertake physical exercise by different stimuli, such as emotional involvement, socialization, and mental and physical wellbeing as well as the achievement and maintenance of a positive self-image.

In this scenario, within the different genes and pathways identified in the brain and shown to be involved in the stimulation or suppression of these behavioral responses, a role has been hypothesized for Fos/DeltaFosB [120] and leptin signaling pathways [121]. In particular, it has been shown that dopamine-mediated neuronal activities in the CNS of females are dependent on estrogens and ER distribution in different brain regions [122,123]. A sex-dependent estrogen mediation of dopamine signaling pathways has been highlighted in females in the striatum and nucleus accumbens, which is linked to reproductive success overall [116].

## 4. Conclusions

CVD is the leading cause of death worldwide and remains a major global economic burden. Recent findings have highlighted the existence of crucial sex/gender-related differences in the prevalence, presentation, management, and outcomes of different CVDs, which are related to differences in fat tissue distribution and different hormonal profiles and fluctuations, such as those during the menstrual cycle, pregnancy, and delivery in women. Moreover, evidence and findings of studies have recently assessed the importance of exercise and physical activity in the reduction of CVD, strongly suggesting the need for physical exercise-based medicine along with a personalized-medicine approach.

We are aware that there are major limitations involved, particularly relating to the absence of well-established protocols to investigate and measure the biological effects of different physical exercise training in the context of CVD, along with the lack/scarcity of preclinical/clinical studies properly addressing the role of gender, other than the one of sex. Nonetheless, we believe that the focus of the present review in addressing a potential sex/gender-specific approach to preventing/managing CVD in women, which is at a disadvantage in the long term due to the clinical standard care, is of great relevance and may be of inspiration for new and innovative research in the field [1,3,8].

**Figure 4:**
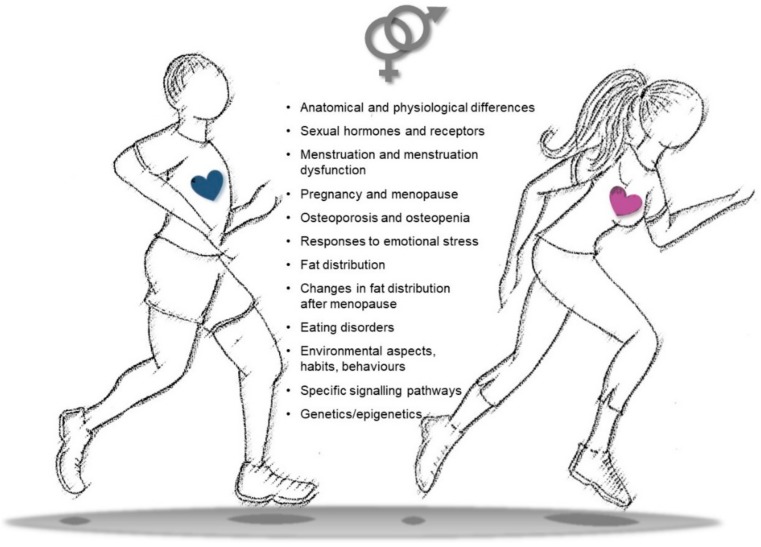
Snapshot of key differences in sex/gender features to be considered in physical activity to improve cardiovascular health, decrease cardiovascular disease (CVD) risk, and settle personalized training/rehabilitative exercise protocols.

Further studies are indeed needed to define if an increase in physical activity/training in women should be defined at appropriate levels, in different intensities, and according to the type of exercise in order to maximize the positive effects on health, CVD prevention, and rehabilitation processes. Similarly, gender-specific aspects should also be taken into account at different ages to identify habits, attitudes, and behaviors that may influence the outcomes of physical exercise. Differences in gender-related imprinting during the early phases of life may indeed become more relevant during adult life, accounting for educational, social, and cultural rules that may influence behaviors, lifestyle, adherence to training schedules, and transgenerational epigenetic inheritance (Figure 4).

Finally, particular attention should be paid in the future to the transgender population. In light of the use of sex hormone treatments, this population will need careful consideration in order to address the real impact of therapies on metabolic profile, CVD risk, and other pathological conditions such as diabetes as well as to establish how tailored physical activity may decrease disease risk.

In conclusion, a better understanding of the genetic and molecular mechanisms driving sex/gender-related differences is needed in order to promote mechanism-based precision medicine protocols and gain insights for the development of sex-specific therapeutic strategies for CVD based on ad-hoc and ad personam physical exercise approaches.

## Abbreviations

CAD	coronary artery disease
CV	cardiovascular
CVD	cardiovascular disease
ERα	estrogen receptor-α
ERβ	estrogen receptor-β
ERs	estrogen receptors
eNOS	endothelial nitric oxide synthase
FMD	flow-mediated dilatation
a-FABP	adipocyte fatty acid-binding protein
IHD	ischemic heart disease
MACE	major adverse cardiac event
MAPK	mitogen-activated protein kinase
NO	nitric oxide
WHO	World Health organization
